# Der deutsche Sydney Swallow Questionnaire: Reliabilität und Validität bei Patienten mit oropharyngealer Dysphagie

**DOI:** 10.1007/s00106-021-01000-9

**Published:** 2021-02-19

**Authors:** J. E. Bohlender, S. Frick, U. Colotto, S. Hotzenköcherle, M. Brockmann-Bauser

**Affiliations:** 1 Abteilung Phoniatrie und Klinische Logopädie, Klinik für Ohren‑, Nasen‑, Hals- und Gesichtschirurgie, Universitätsspital Zürich, Universität Zürich, Frauenklinikstrasse 24, 8091 Zürich, Schweiz; 2 Universität Zürich, Zürich, Schweiz

**Keywords:** Schluckstörung, Oropharyngeale Dysphagie, Gesundheitsbezogene Lebensqualität, Funktioneller Gesundheitsstatus, Patient-Reported Outcome Measure, Dysphagia, Oropharyngeal dysphagia, Functional health status, Health-related quality of life (HRQoL), Patient-reported outcome measures

## Abstract

**Hintergrund:**

Der Sydney Swallow Questionnaire (SSQ) ist ein patientenbasierter Fragebogen zur Erhebung von subjektiven Beschwerden oropharyngealer Schluckstörungen unterschiedlicher Ursachen, mit starker inhalts-, konstrukt-, diskriminativer und prädiktiver Validität.

**Ziel der Arbeit/Fragestellung:**

Ziel dieser Arbeit war die Überprüfung der Reliabilität und Validität der deutschen Fassung des Sydney Swallow Questionnaire (SSQ-G).

**Material und Methode:**

In einer Kreuzvalidierungsstudie füllten 48 erwachsene deutschsprachige Patienten (12 Frauen/36 Männer) mit neurogen (*n* = 16), strukturell (*n* = 16) und funktionell (*n* = 16) bedingten Schluckstörungen den deutschen SSQ‑G und MD Anderson Dysphagia Inventory (MDADI) aus. Die Reliabilität des SSQ‑G wurde anhand der internen Konsistenz mittels Cronbach‑α berechnet. Die Überprüfung der Kriterien- und der Konstruktvalidität erfolgte durch eine Kreuzvalidierung mittels Spearman-Korrelationskoeffizient.

**Ergebnisse:**

Die interne Konsistenz des SSQ‑G war mit Cronbach-α = 0,94 exzellent. Die SSQ-G-Fragen 1 und 17 wiesen mit MDADI-Frage 1 einen moderat signifikanten bzw. hochsignifikanten Korrelationskoeffizienten von −0,43 und −0,45 auf (*p* < 0,5; *p* < 0,001). Zwischen Fragen 8, 11 und 12 des SSQ‑G und Fragen 7, 13 und 10 des MDADI lag mit Korrelationen von −0,48 bis −0,55 ein mittlerer bis starker hochsignifikanter Zusammenhang vor (*p* < 0,001). Somit wurden die Reliabilität, die Kriteriums- und Konstruktvalidität statistisch bestätigt.

**Schlussfolgerung:**

Mit der deutschen Version des SSQ (SSQ-G) können funktionsspezifische Schluckbeschwerden reliabel und valide erfasst werden. In Kombination mit Fragenbögen zur symptomspezifischen Lebensqualität wie dem MDADI ist somit eine differenziertere klinische Analyse von Schluckbeschwerden möglich.

**Zusatzmaterial online:**

Die Online-Version dieses Beitrags (10.1007/s00106-021-01000-9) enthält die deutsche Fassung des Sydney Swallow Questionnaire. Beitrag und Zusatzmaterial stehen Ihnen auf www.springermedizin.de zur Verfügung. Bitte geben Sie dort den Beitragstitel in die Suche ein, das Zusatzmaterial finden Sie beim Beitrag unter „Ergänzende Inhalte“.

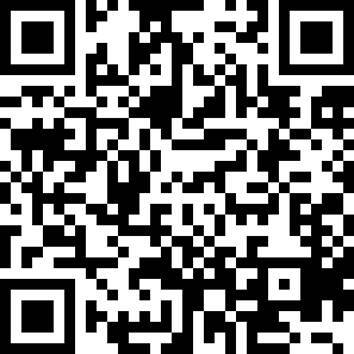

## Abstract (en)

**Background:**

The Sydney Swallow Questionnaire (SSQ) is a self-report inventory assessing subjective symptoms of oropharyngeal dysphagia with strong content, construct, discriminant, and predictive validity and test–retest reliability in a range of patient populations.

**Objective:**

The main aim of this work was to assess the validity and reliability of the German version of the SSQ (SSQ-G).

**Materials and methods:**

In a cross-validation study, 48 adult German-speaking patients (12 women, 36 men) with neurogenic (*n* = 16), structural (*n* = 16), and functional (*n* = 16) oropharyngeal dysphagia were assessed with the SSQ‑G and the MD Anderson Dysphagia Inventory (MDADI). Cronbach’s α was applied to assess the reliability. Criteria and construct validity were investigated using the Spearman correlation coefficient.

**Results:**

With Cronbach’s α = 0.94, the internal consistency of the SSQ‑G was excellent. The SSQ‑G questions 1 and 17 showed a moderately significant and highly significant correlation coefficient of −0.43 and −0.45, respectively, with MDADI question 1 (*p* < 0.5, *p* < 0.001). Between questions 8, 11, and 12 of the SSQ‑G and questions 7, 13, and 10 of the MDADI, coefficients of −0.48 to −0.55 showed a moderate to strong highly significant correlation (*p* < 0.001). Thus, the reliability and criterion and construct validity were statistically confirmed.

**Conclusion:**

The German version of the SSQ (SSQ-G) allows a reliable and valid assessment of functional swallowing difficulties. In combination with questionnaires on symptom-specific quality of life, such as the MDADI, a more differentiated clinical analysis of swallowing problems is thus possible.

Patienten mit einer oropharyngealen Dysphagie weisen vielfältige Symptome auf, welche von einem zervikalen Globusgefühl bis hin zu einer objektivierbaren schwerwiegenden Schluckunfähigkeit reichen können [26]. Die zugrunde liegenden Ursachen sind vielfältig und erklären dabei häufig nur teilweise die Art und den Grad der Funktionseinbußen beim Schlucken. Mit dem hier vorgestellten deutschen Sydney Swallow Questionnaire (SSQ-G) können die subjektiv erlebten funktionellen Einschränkungen und Beschwerden bei Patienten mit einer oropharyngealen Dysphagie standardisiert erfasst werden. In dieser Arbeit wurde die Reliabilität und Validität des SSQ-G anhand von Patienten mit neurogen, strukturell und funktionell bedingten Schluckstörungen überprüft.

Im klinischen Alltag haben sich bei der weiterführenden Diagnostik von Dysphagiebeschwerden instrumentelle Abklärungsverfahren, mittels derer Art, Ätiologie und Schweregrad der Beeinträchtigung beurteilt werden können, etabliert. Mit den beiden instrumentellen Verfahren FEES® („flexible endoscopic evaluation of swallowing“) [18] und VFSS („videofluoroscopic swallowing study“) kann unabhängig von der Ursache eine Penetration und Aspiration nachgewiesen, und deren Schweregrad beispielsweise mittels Penetrations-Aspirations-Skala nach Rosenbek (PAS) bestimmt werden [13, 27].

Jedoch wird der Schweregrad einer Schluckstörung und die damit einhergehenden subjektiven Einschränkungen trotz gleicher Erkrankung unabhängig von der klinischen Diagnostik von Patient zu Patient unterschiedlich erlebt und bewertet. Diese individuelle patientenzentrierte Perspektive als eigenständiges Maß spiegelt sich im Konzept der subjektiven gesundheitsbezogenen Lebensqualität (Health-Related Quality of Life [HRQoL]) oder dem funktionellen Gesundheitsstatus (Functional Health Status [FHS]) wider. Die HRQoL beschreibt idealerweise fünf Dimensionen von Wohlbefinden und Funktionsfähigkeit (physisch, emotional, mental, sozial und verhaltensbezogen) aus der Sicht der betroffenen Patienten. Im Gegensatz hierzu beschreibt der funktionelle Gesundheitsstatus den individuell erlebten und bewerteten Schweregrad eines spezifischen Symptoms, beispielsweise eine oropharyngeale Dysphagie. Die gesundheitsbezogene Lebensqualität und der FHS [23] werden mithilfe von spezifischen Fragebögen (Patient-Reported Outcome Measures [PROM]) standardisiert erfasst. Diese patientenzentrierte Perspektive wird neben objektiven klinischen Daten als integraler Bestandteil eines zeitgemäßen klinischen Behandlungskonzepts verstanden. So können Fragebögen zusätzlich zur Beurteilung von Behandlungsqualität und -kosten sowie zur Optimierung von Versorgungspfaden herangezogen werden. Im deutschsprachigen Raum gibt es derzeit nur drei validierte dysphagiespezifische Fragebögen mit unterschiedlichen psychometrischen Eigenschaften (Tab. 1). Der SWAL-QOL [17] und MD Anderson Dysphagia Inventory (MDADI) [3] erfassen die gesundheitsbezogene Lebensqualität. Der kürzlich ins Deutsche übersetzte „Eating Assessment Tool-10“ (Eat-10) hingegen erfüllt die Kriterien eines funktionellen Gesundheitsstatus-Fragenbogens [30]. Mit der hier vorgestellten deutschen Version des SSQ (SSQ-G) [15], die in Anlehnung an eine transkulturelle Übersetzung nach Beaton [4] erstellt wurde, ist nun ein weiterer Fragebogen verfügbar, der primär funktionelle Beschwerden bei einer oropharyngealen Dysphagie erfasst (elektronisches Zusatzmaterial online).

**Table 1:** 

Name	Autor (Original/deutsche Übersetzung)	Anzahl Items, Unterskalen und Antwortmöglichkeiten	Psychometrische Eigenschaften und Unterskalen	Untersuchte Gruppe (*N*), Durchschnittsalter und Parameter
SWAL-QOL (Swallowing Quality of Life Questionnaire)	Mc Horney CA et al. (2000, 2002) [20–22] Deutsche Übersetzung: Kraus EM et al. (2018) [17]	44 Items 10 Unterskalen plus Symptomskalierung 1–5-Punkte-Likert-Skala	1. Anstrengung 2. Verlangen nach Essen 3. Zeitdauer zum Essen 4. Essensbeschränkung 5. Kommunikation 6. Angst 7. geistige Gesundheit 8. Alltagsfunktion 9. Schlaf 10. Ermüdung 11. Symptomhäufigkeit	*N* = 386 [Phase III] [21] Kontrollgruppe *N* = 40
				Unterschiedliche strukturelle und neurogene Dysphagien
				Durchschnittsalter: 66,1 J
				Interne Konsistenz mit Cronbach-α-Koeffizienten von 0,79 bis 0,91
MDADI (MD Anderson Dysphagia Inventory)	Chen AY et al. (2001) [7] Deutsche Übersetzung: Bauer F et al. (2010) [3]^a^	4 Dimensionen 20 Items 1–5- Punkte-Likert-Skala	Global (1 Item) emotional (6 Items) funktionell (5 Items) physisch (8 Items)	*N* = 100 Patienten mit benignen und malignen Kopf-Hals-Tumoren
				Durchschnittsalter: 58 J
				Interne Konsistenz mit Cronbach-α-Koeffizienten von 0,85 bis 0,93
				Koeffizienten der Test-Retest-Reliabilität: 0,69–0,88
				Spearman-Korrelationskoeffizienten zwischen MDADI-Unterskalen und SF-36-Unterskalen bestätigte eine Konstruktvalidität
SSQ (Sydney Swallow Questionnaire)	Wallace KL et al. (2000) [29]^b^ [Self-report Symptom Inventory] Dwivedi RC et al. (2010) [11] Deutsche Übersetzung: Hotzenköcherle S (2011) [15]	17 Items in 3 Unterskalen visuelle Analogskala (0–100) 5‑Punkte-Skala (1 Item)	Global physisch emotional	(A) Wallace KL et al. Neurogene Dysphagien *N* = 48 Durchschnittsalter 58 J Zenker-Divertikel (präoperativ) *N* = 11 Durchschnittsalter 66 J Kontrollgruppe *N* = 19
				(B) Dwivedi RC et al. orale und oropharyngeale Karzinome^c^ insgesamt *N* = 54 davon *N* = 16 (orale Karzinome) *N* = 38 (oropharyngeale Karzinome) Durchschnittsalter insgesamt 58,6 J Reliabilität Cronbach-α-Wert von 0,95. Konstruktvalidität mit Spearman-Korrelationskoeffizienten von 0,46–0,77
EAT-10 (Eating Assessment Tool-10)	Belafsky PC et al. (2008) [5] Deutsche Übersetzung: Zaretsky et al. (2018) [30]	10 symptombezogene Fragen 5‑Punkte-Likert Skala (0 = keine, 4 = große Probleme)	Dysphagiespezifische Fragen, die vom Autor keine weitere Zuordnung erhalten	(I) Gesunde Personen (*N* = 100; 53 M, 47 F); Durchschnittsalter insgesamt 48 J
				(II) Stimm- und Schluckstörungen (*N* = 235; 127 M, 108 F) Durchschnittsalter insgesamt 62 J
				(III) Patienten mit Schlucktherapie (*N* = 46; 21 M, 25 F); Durchschnittsalter insgesamt 65 J Interne Konsistenz mit Cronbach-α: 0,96 (hohe Konsistenz) Test-Retest-Reliabilität exzellent (0,72–0,91)

^a^Deutsche Übersetzung verwendet das Akronym ADI‑D (Anderson-Dysphagia-Inventory, deutsche Version) [3]

^b^SSQ ist identisch mit dem ursprünglichen patientenzentrierten symptomspezifischen Fragebogen (Self-report Symptom Inventory) von Wallace [29]

^c^Wird bei Patel DA als SSQ-hn [23] genannt

Die Originalversion des SSQ [29], ursprünglich als Self-report Symptom Inventory bezeichnet, wurde anhand von 45 Patienten mit neurogenen oropharyngealen Dysphagien von KL Wallace et al. entwickelt und validiert. Der ursprüngliche Self-report Symptom Inventory wurde 2010 von RC Dwivedi et al. als SSQ-Fragebogen in die Fachliteratur eingeführt und an Patienten mit Kopf-Hals-Karzinomen validiert [11]. Die angloamerikanische Version des SSQ ist als dysphagiespezifischer Fragebogen an Patienten mit neurogenen, strukturellen und altersbedingten Schluckbeschwerden validiert worden. Hier zeigte sich, dass der SSQ bei verschiedenen Ursachen krankheitsbezogene subjektive Symptome einer oropharyngealen Dysphagie reliabel, valide und sensitiv erfasst [10, 11, 28, 29]. Mittlerweile liegen eine schwedische [1] und eine französische [2] Übersetzung des SSQ vor. Ziel der vorliegenden Arbeit war die Überprüfung der Reliabilität und Validität der deutschen Fassung des SSQ im Vergleich zu dem international gängigen MDADI in der deutschsprachigen Version.

## Material und Methode

### Studiendesign und Einschlusskriterien

In einer prospektiven Kreuzvalidierungsstudie füllten 48 erwachsene Patienten (12 Frauen und 36 Männer) im Alter von 29–89 Jahren (Median: 72,0 Jahre, SD ± 14,0) zwischen August 2013 und Juni 2014 den SSQ‑G und die deutsche Fassung des MDADI im Rahmen der Dysphagie-Sprechstunde aus.

Alle Patienten wurden zur weiterführenden Untersuchung bei dysphagischen Beschwerden an der Abteilung Phoniatrie und Klinische Logopädie der ORL-Klinik des Universitätsspitals Zürich angemeldet und nahmen freiwillig an dieser Studie teil. Die Diagnosen wurden anhand einer vollständigen Dysphagie-Untersuchung mit Anamnese, klinischer Untersuchung und flexibler endoskopischer Evaluation des Schluckvorgangs nach dem Langmore-Protokoll (FEES®) [14, 16, 18] gestellt. Um eine möglichst repräsentative Gruppe von Patienten in die Validierung mit einzubeziehen, wurden je 16 Patienten mit einer neurogen, strukturell und funktionell bedingten Schluckstörung eingeschlossen. (Funktionell wird hier im Sinne einer „Ausschlussdiagnose“ verwendet und umfasst dysphagische Patienten, die keine neurogenen oder strukturellen Alterationen aufweisen).

Alle Patienten mussten deutschsprachig, über 18 Jahre alt und in der Lage sein, den Bogen selbstständig auszufüllen. Ausgeschlossen wurden Patienten, welche vollumfänglich über eine Nasogastralsonde; PEG oder PEJ oder anderweitig parenteral ernährt wurden. Die vorliegende Studie wurde von der zuständigen Ethikkommission (Referenz-Nummer KEK ZH 2011-0210 und KEK ZH 2013-0226) genehmigt.

### Kreuzvalidierung der ersten deutschen Version

Für die Kreuzvalidierung wurden der SSQ-G [15] und die deutsche Version des MD Anderson Dysphagia Inventory (ADI-D) [3] verwendet. Alle Patienten mussten mindestens 16 Fragen im SSQ‑G und mindestens 18 Fragen im MDADI beantwortet haben.

#### Sydney Swallow Questionnaire

**Figure 1:**
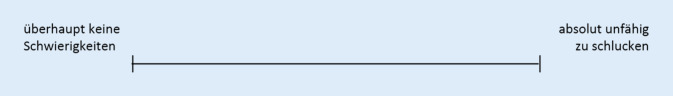

Der SSQ‑G erhebt in 17 Fragen subjektive Schluckschwierigkeiten in Bezug auf die Konsistenz des Bolus, Einschränkungen in der oralen und pharyngealen Phase einschließlich des velopharyngealen Abschlusses sowie Anzeichen einer Penetration, Aspiration oder nasalen Regurgitation. Die Fragen können den drei Unterskalen global, physisch bzw. schluckbezogene Lebensqualität zugeordnet werden: Frage 1 global, Fragen 2–16 physisch und Frage 17 schluckbezogene Lebensqualität ([29]; Tab. 2). Alle Fragen werden auf einer 100 mm langen visuellen Analogskala mit einem einzigen Kreuz beantwortet (Abb. 1).

**Table 2:** 

Item Nr./Skala	Inhalt	Originalversion SSQ (Wallace et al. 2010) [29]	Deutsche Version SSQ‑G (S. Hotzenköcherle 2011) [15]
1 Global	Global	*How much difficulty do you have swallowing at present?*	Wie groß sind Ihre Schwierigkeiten in letzter Zeit beim Schlucken?
2 Physisch	Konsistenz	*How much difficulty do you have swallowing THIN liquids? (e. g.: tea, softdrink, beer, coffee)*	Wie viel Schwierigkeiten bereitet Ihnen das Trinken von dünnflüssigen Getränken wie Tee, Softdrinks, Bier oder Kaffee?
3 Physisch		*How much difficulty do you have swallowing THICK liquids? (e. g.: milkshakes, soups, custard)*	Wie viel Schwierigkeiten bereitet Ihnen das Schlucken von dickflüssigen Getränken wie Milkshake, Suppe oder Vanillecreme?
8 Physisch	Schluckassoziierte Symptome	*Do you have any difficulty starting a swallow?*	Haben Sie Schwierigkeiten bei Beginn des Schluckens?
12 Physisch	Essensdauer	*How long does it take you to eat an average meal?*	Wie lange benötigen Sie für eine durchschnittliche Mahlzeit? Bitte kreuzen Sie eine Möglichkeit an: □ Weniger als 15 min □ Ungefähr 15–30 min □ Ungefähr 30-45 min □ Ungefähr 45–60 min □ Mehr als 60 min □ Ich kann überhaupt nicht schlucken
13 Physisch	Schluckassoziierte Symptome	*When you swallow does food or liquid go up behind your nose or come out of your nose?*	Gelangen Flüssigkeiten oder Nahrungsmittel beim Schlucken in die Nase oder kommen aus der Nase heraus?
16 Physisch	Subjektiver Schweregrad	*How do you rate the severity of your swallowing problem today?*	Wie hoch schätzen Sie den Schweregrad des Schluckproblems heute ein?
17 Emotional	Lebensqualität	*How much does your swallowing problem interfere with your enjoyment or quality of life?*	Wie sehr behindert Ihr Schluckproblem Ihre Lebensfreude oder Lebensqualität?

Ein Kreuz auf der linken Seite bei 0 mm entspricht der Merkmalsausprägung „nicht vorhanden/ausgeprägt“ und rechtsseitig bei 100 mm „maximal ausgeprägt“. Einzige Ausnahme ist Frage Nr. 12 „Wie lange benötigen Sie für eine Mahlzeit?“ mit einer numerischen Auswertungsskala von 0 bis 5. Je höher der Wert, desto ausgeprägter sind die subjektiven schluckspezifischen Beschwerden. Zur Auswertung wird die Antwort auf Frage Nr. 12 mit 20 multipliziert, für ein maximales Gesamtergebnis von 1700 Punkten bei einer maximal ausgeprägten subjektiven Schluckstörung [29].

#### MD Anderson Dysphagia Inventory

In der vorliegenden Studie wurde die deutsche Version des MDADI als Goldstandard verwendet [3]. Er besteht aus 20 Fragen, welche den globalen (Frage 1, Zusammenfassungsfrage), emotionalen (Fragen E1 – 6: 2, 5, 6, 8, 12, 18), funktionellen (Fragen F1 – 5: 3, 9, 14, 15, 20) und physischen Unterskalen (Fragen P1 – 8: 4, 7, 10, 11, 13, 16, 17, 19) zugeordnet werden. Die Fragen werden auf einer fünfstufigen Likert-Skala („stimme voll und ganz zu“ bis hin zu „stimme auf keinen Fall zu“) beantwortet.

Ausnahmen sind Fragen 5 (E7) und 15 (F2), welche eine umgekehrte Skalierung aufweisen. Für die Auswertung im Rahmen dieser Studie wurde diese umgekehrt und somit den anderen Antworten numerisch angepasst. Danach wurde der jeweils gesamte Mittelwert für die Unterskalen emotional, funktionell und physisch sowie das kombinierte Ergebnis dieser Unterskalen berechnet. Alle Mittelwerte wurden mit 20 multipliziert, sodass ein Gesamtergebnis zwischen 20 (hohe Einschränkung) und 100 (keine Einschränkung) entstand. Je höher dieser Wert ausfiel, desto geringer war somit der Ausprägungsgrad der subjektiven Beschwerden [7].

### Statistik

Das Alter der Patienten sowie die Ergebnisse der einzelnen Fragen wurden in einem Excel-Arbeitsblatt (Excel 11; Fa. Mac Corp., WA, USA) tabelliert. Die statistischen Analysen wurden mittels SPSS 22 (Fa. SPSS Inc., Chicago/IL, USA) durchgeführt. Zunächst wurde die Verteilung der Mittelwerte mit Standardabweichung sowie die tatsächliche Spanne der Gesamtergebnisse und ihrer Unterskalen für beide Fragebögen berechnet bzw. exzerpiert. Da der MDADI eine Ordinalskalierung aufweist, wurden zusätzlich Medianwerte zur besseren Vergleichbarkeit ermittelt. Mittels Boxplots wurde die Verteilung auf Ausreißer überprüft.

Für die Reliabilitäts- und Validitätsanalyse des SSQ‑G wurden die Fragen in drei Unterskalen eingeteilt: globale Schluckfunktion (Frage 1), funktionelle und physiologische Schluckfunktion (Fragen 2–16) und schluckbezogene Lebensqualität (Frage 17). Die Reliabilität wurde anhand der internen Konsistenz mittels Cronbach‑α (Alpha) bestimmt. Eine hohe durchschnittliche Korrelation aller Items eines Tests, bei einem akzeptablen bis exzellenten Cronbach-α-Wert von > 0,7, bestätigt die interne Konsistenz [8].

Die Validität des SSQ‑G wurde mittels Kreuzvalidierung mit dem MDADI untersucht. Da eine transkulturelle Übersetzung der Originalversion vorgenommen wurde, wurde die *Inhaltsvalidität* analog zur Originalversion als gegeben angenommen [15]. Die *Konstruktvalidität* des SSQ‑G wurde mittels einer bivariaten Korrelation nach Spearman von drei Fragen des SSQ‑G mit drei inhaltlich sehr ähnlichen Fragen des MDADI bestimmt. Verglichen wurden SSQ-G-Frage 1 und MDADI-Frage 1, SSQ-G-Frage 8 und MDADI-Frage 7, SSQ-G-Frage 11 und MDADI-Frage 13 sowie SSQ-G-Frage 12 und MDADI-Frage 10.

Die Kriteriumsvalidität wurde mittels Spearman-Korrelationskoeffizient der globalen Schluckfunktion (Frage 1 des SSQ-G), der physiologischen Schluckfunktion (Fragen 2–16 des SSQ-G) und der dysphagiebezogenen Lebensqualität (Frage 17 des SSQ-G) mit den globalen (Frage 1 des MDADI) und den physischen Unterskalen des MDADI (Fragen 4, 7, 10, 13, 16, 17, 19) überprüft. Ergebnisse von > 0,60 wurden als starke Korrelation, Werte von 0,40 bis 0,60 als moderate und Werte < 0,40 als schwache Korrelation interpretiert, bei einem Signifikanzniveau von *p* < 0,5 [12].

## Ergebnisse

### Verteilung der Mittel- und Medianwerte

Der Mittelwert für das Gesamtergebnis des SSQ‑G betrug 536,9 (SD 314,3) und der Medianwert 596,0 bei einer Spannweite von minimal zwei zu maximal 1247 Punkten (Tab. 3). Für den MDADI lag ein Mittelwert von 50,4 (SD 15,7), ein Medianwert von 50,0 und eine Spannweite von 24,2 bis 96,8 für den kombinierten Gesamtwert (Fragen 2–20) vor (Tab. 4). Eingeschlossen wurden ein unvollständiger SSQ‑G mit einer fehlenden Antwort, sieben MDADI-Fragebögen mit einer und zwei MDADI-Fragebögen mit zwei fehlenden Antworten. Die häufigsten nicht beantworteten Fragen des MDADI waren F5 („Wegen meiner Schluckbeschwerden musste ich Einkommenseinbußen hinnehmen“) und P6 („Das Schlucken fällt mir sehr schwer“). Beim MDADI zeigte sich in der Verteilung der Ergebnisse eine Person mit im Vergleich zur Gruppe überdurchschnittlich stark ausgeprägten Beschwerden gemäß MDADI („Ausreißer“). Für die Ergebnisse des SSQ‑G lag kein Ausreißer vor.

**Table 3:** 

SSQ‑G	Global (Frage 1)	Physisch (Fragen 2–16)	Lebensqualität (Frage 17)	Gesamt (Fragen 1–17)
*Mittelwert*	38,0	491,9	42,3	536,9
*Median*	45,0	485,0	45,0	596,0
*SD*	22,4	281,7	35,5	314,3
*Min*	0,0	2,0	0,0	2,0
*Max*	95,0	1163,0	98,0	1247,0

**Table 4:** 

MDADI	Global (Frage 1)	Emotional (Fragen 2, 5, 6, 8, 12, 18)	Funktionell (Fragen 3, 9, 14, 15, 20)	Physisch (Fragen 4, 7, 10, 11, 13, 16, 17, 19)	Kombiniert (Fragen 2–20)
*Mittelwert*	61,2	52,9	46,7	55,2	50,4
*Median*	80,0	53,3	46,0	60,0	50,0
*SD*	27,2	18,2	17,1	17,9	15,7
*Min*	20,0	20,0	20,0	20,0	24,2
*Max*	100,0	100,0	100,0	95,0	96,8

### Reliabilität und Validität

Die interne Konsistenz (Reliabilität) des SSQ‑G wurde durch ein von Cronbach-α = 0,94 bestätigt. Diese lag minimal höher als beim MDADI mit α = 0,90.

Bei drei inhaltlich ähnlichen Fragen der Fragebögen SSQ‑G (Fragen 8, 11 und 12) und MDADI (Fragen 7, 13 und 10) lag mit Korrelationskoeffizienten von −0,48 bis −0,55 ein mittlerer bis stark ausgeprägter hochsignifikanter Zusammenhang vor (*p* < 0,001, Tab. 5). Somit wurde die Konstruktvalidität statistisch bestätigt.

**Table 5:** 

	MDADI		
SSQ‑G	P6 (Frage 7)	P7 (Frage 10)	P8 (Frage 13)
*Frage 8*	−0,54**	–	–
*Frage 11*	–	–	−0,48**
*Frage 12*	–	−0,55**	–

** kennzeichnet hochsignifikante Ergebnisse (*p* < 0,001)

Zwischen der Zusammenfassungsfrage (global, Frage Nr. 1) und Frage 17 des SSQ‑G und der Frage 1 des MDADI (global) lag jeweils ein moderater signifikanter und hochsignifikanter Korrelationswert von −0,43 bzw. −0,45 und -0,55 vor (*p* < 0,05 und *p* < 0,001; Tab. 6). Die Unterskalen SSQ‑G physisch und MDADI physisch wiesen eine stark ausgeprägte hochsignifikante Korrelation von −0,67 auf (*p* < 0,001; Tab. 6). Die Kriteriumsvalidität des SSQ‑G wurde somit bestätigt.

**Table 6:** 

	MDADI	
SSQ‑G	Global (Frage 1)	Physisch (Fragen 4, 7, 10, 11, 13, 16, 17, 19)
*Total (alle Items)*	−0,55**	−0,697**
*Physisch (Fragen 2–16)*	–	−0,67**
*Global (Frage 1)*	−0,43*	−0,55**
*Lebensqualität (Frage 17)*	−0,45*	–

** kennzeichnet hochsignifikante (*p* < 0,001) und * signifikante Ergebnisse (*p* < 0,5)

## Diskussion

Die hier vorgestellte deutsche Version des Sydney Swallow Questionnaire (SSQ-G) ist ein reliables und valides Messinstrument in Form eines Fragebogens, das subjektive funktionelle Einschränkungen bei einer oropharyngealen Dysphagie unterschiedlichster Ursachen systematisch und standardisiert aus der Sicht von Patienten erfasst. Es zeigte sich eine exzellente interne Konsistenz des SSQ‑G mit Cronbach-α = 0,94. Außerdem konnte die Reliabilität, aber auch die Kriteriums- und Konstruktvalidität im Vergleich zum MDADI statistisch bestätigt werden.

### Reliabilität und Validität des SSQ-G

Die Reliabilitätsüberprüfung durch Bestimmung der internen Konsistenz ergab im SSQ‑G einen Cronbach-α-Wert von 0,94. Dieses Ergebnis deckt sich mit der ermittelten internen Konsistenz des Cronbach‑α von 0,95 mit der Originalversion von Dwivedi et al. [11]. Ein vergleichbarer Wert wird in der französischen Version SSQ‑f mit 0,956 angegeben [2]. Für die schwedische SSQ-Version, welche an 20 Patienten validiert wurde, ist kein Cronbach-α-Wert angegeben [1]. Somit zeigt der SSQ‑G im Vergleich zur die Originalversion und der SSQ‑f eine hohe Übereinstimmung in der internen Konsistenz.

Für die Konstruktvalidität berichten Dwivedi et al. [11] über Korrelationswerte von 0,46 bis 0,77, welche im deutschsprachigen SSQ‑G mit Werten von −0,48 bis −0,557 teilweise bestätigt werden konnten. In der französischen und schwedischen Version des SSQ erfolgte die Berechnung der Konstruktvalidität anhand des MDADI nicht. Für den SSQ‑G lag eine stark ausgeprägte hochsignifikante Korrelation zwischen dem totalen SSQ‑G und der „physischen“ Dimension des SSQ‑G und den MDADI-Unterskalen „physisch“ und „global“ vor. Somit entspricht die Kriteriumsvalidität weitgehend den Ergebnissen der angloamerikanischen Publikation von Dwivedi et al. [11]. Im Vergleich dazu war die Korrelation zwischen der Lebensqualitätsfrage des SSQ‑G gegenüber dem MDADI „global“ lediglich moderat signifikant. Die unterschiedlichen Ergebnisse innerhalb der Unterskalen können möglicherweise mit der kleinen und heterogenen Studienpopulation erklärt werden. Interessanterweise ließen sich in einer 2017 erschienenen Publikation [25], die anhand von 89 Patienten mit Kopf-Hals-Tumoren SSQ- und MDADI-Daten miteinander korrelierte, bis auf die SSQ-Frage zur Lebensqualität (Frage 17) teilweise nur schwache Korrelationen zwischen den einzelnen Unterskalen nachweisen. Die Autoren argumentieren, ohne dabei die signifikanten Ergebnisse von Dwivedi et al. zu diskutieren, dass die fehlende Korrelation auf die unterschiedliche Ausrichtung der Fragebögen, funktionsspezifisch versus Lebensqualität, zurückzuführen sei. Eine Clusteranalyse, basierend auf dem Vergleich von MDADI zum SSQ, konnte die insgesamt aus 89 Patienten bestehende Gesamtgruppe in weitere drei Gruppen differenzieren. So zeigte beispielweise eine Gruppe von Patienten lediglich eine moderate Schluckbeeinträchtigung bei einer gleichzeitig deutlich eingeschränkten dysphagiebezogenen Lebensqualität an. Die Autoren empfehlen in ihrer Schlussfolgerung den Gebrauch beider Fragebögen bei Patienten mit Kopf-Hals-Tumoren, da diese einen unterschiedlichen Fokus aufweisen.

Eine vergleichbare Clusteranalyse lässt sich bei der kleineren Fallzahl in unserer Gruppe nicht durchführen. Es stellt sich dennoch die Frage, ob die klinische Beobachtung, dass zwischen symptombezogener Lebensqualität und der funktionellen Einschränkung ein Widerspruch bestehen kann, nicht nur bei strukturellen Dysphagien, sondern auch bei Patienten mit neurogenen oder funktionellen Dysphagien in Zukunft stärker berücksichtigt werden sollte. Dieses klinische Dilemma findet sich auch bei objektiv erhobenen Ergebnissen einer instrumentellen Dysphagiediagnostik (z. B. PAS) im Verhältnis zu der subjektiven dysphagiebezogenen Lebensqualität (z. B. MDADI) [24].

Limitierend muss im Hinblick auf diese Studie allerdings festgehalten werden, dass die untersuchte Fallzahl eher niedrig war und die Studienpopulation unterschiedliche Ursachen einer oropharyngealen Dysphagie aufwiesen. Es lassen sich daher keine spezifischeren Aussagen ableiten. Zukünftig sollten daher größere und gut phänotypisierte Patientengruppen untersucht werden, um beispielsweise mithilfe von Cut-off-Werten Risikopatienten zu identifizieren.

### Klinische Anwendbarkeit

Der SSQ‑G erfüllt mit 17 Fragen die Kriterien eines klar verständlichen und lesbaren Fragebogens, der keine überdurchschnittliche Lesekompetenz der Patienten oder Hilfe beim Ausfüllen erfordert [31]. So ist das Problem einer eingeschränkten Verständlichkeit und Lesbarkeit beispielsweise beim SWAL-QoL [20] bekannt. Im Niederländischen besteht deswegen gleichzeitig neben der seit 2009 validierten Übersetzung der angloamerikanischen Originalversion [6] eine verständlichere adaptierte Version des SWAL-QoL (Adjusted DSWAL-QoL) [19].

Das Ausfüllen des SSQ-G-Fragebogens nimmt in der Regel weniger als zehn Minuten [15] in Anspruch, die englischsprachige Originalversion und Wallace et al. gehen sogar von einer durchschnittlichen Bearbeitungszeit von nur fünf Minuten aus [10]. Ergänzend zu diesen testökonomischen Gesichtspunkten wird zudem die Auswertungszeit des SSQ‑G aus der Perspektive des medizinischen Personals lediglich mit durchschnittlich drei Minuten [15] veranschlagt. Somit ist der Fragebogen im klinischen Alltag gut anwendbar.

Mit dem SSQ‑G werden primär aus Sicht der Patienten die subjektiven funktionellen Aspekte einer Schluckbeeinträchtigung erfasst. Die gesundheits- oder symptomspezifische Lebensqualität einer oropharyngealen Dysphagie werden beispielsweise besser mit dem MDADI abgebildet. Auch wenn bis heute keine einheitlichen Vorschläge existieren, um das komplexe Symptom einer oropharyngealen Dysphagie zu erfassen, bietet sich wie beim ELS-Protokoll für Stimmstörungen [9] ein multidimensionaler Zugang bei Schluckstörungen (Tab. 7) an. So empfiehlt sich bei Verdacht auf Schluckstörungen zunächst die Anwendung von dysphagiespezifischen Fragebögen, anschließend erfolgt eine Anamnese mit klinischer Untersuchung (Schritt 2) und ggf. ein Screening (Schritt 3), und erst im 4. Schritt wird eine instrumentelle Diagnostik mittels FEES® (oder VFSS) durchgeführt. Bei Bedarf können ergänzend weiterführende Untersuchungen veranlasst werden (Schritt 5).

**Table 7:** 

Untersuchungsschritt	Methode
1. Dysphagiespezifische Fragebögen mit funktionellen und QoL-Dimensionen	SSQ‑G MDADI DHI
2. Anamnese und klinische Untersuchung	–
3. Screening	z. B.: Gugging Swallowing Screen (GUSS), 100 ml Water Swallow Test (WST)
4. Assessment mittels instrumenteller Diagnostik	FEES® VFSS
5. Bei Bedarf: Weiterführende Untersuchungen	z. B. HRM (High Resolution Manometry)

## Fazit für die Praxis

- Der SSQ‑G weist bei den hier untersuchten Patienten mit Schluckstörungen neurogener, struktureller und funktioneller Ursache eine hohe interne Konsistenz und somit Reliabilität auf. Es liegt eine mittlere bis starke Konstruktvalidität im Vergleich zum MDADI vor.
- Der SSQ‑G bietet sich vor allem für die subjektive Evaluation der funktionellen Beeinträchtigung bei oropharyngealer Dysphagie an.
- Der funktionell ausgerichtete SSQ‑G erlaubt in Kombination mit dysphagiespezifischen Fragenbögen, die auf die symptomspezifische Lebensqualität fokussieren (wie bspw. MDADI oder DHI), eine differenziertere klinische Analyse.

## Supplementary Information




