# Supplementary material for: The German Sydney Swallow Questionnaire: Reliability and validity in patients with oropharyngeal dysphagia
Source: HNO. 2021 Feb 19;69(12):969–77. [Article in German] doi: 10.1007/s00106-021-01000-9 (PMC8613080; doi:10.1007/s00106-021-01000-9)
Supplement: Supplementary file 1 [file 106_2021_1000_MOESM1_ESM.pdf]

USZ Universitäts Spital Zürich
Klinik für Ohren-, Nasen-,
Hals- und Gesichtschirurgie

Prof. Dr. med. A. Huber
Klinikdirektor

Abteilung Phoniatrie und Klinische Logopädie
KD Dr. med. J. Bohlender

Universitätsspital Zürich
Klinik für Ohren-, Nasen-,
Hals- und Gesichtschirurgie
Frauenklinikstr. 24
CH-8091 Zürich

Sekretariat +41-44-255 5830
Telefax +41-44-255 4424
E-Mail: Joerg.Bohlender@usz.ch

Sydney-Schluck-Fragebogen

Name: ....
Geburtsdatum: ....
Geschlecht: □ weiblich □ männlich
Datum: ....

Dieser Fragebogen wurde entwickelt, damit wir den Schweregrad Ihrer Schluckstörung ermitteln können. Er ist nicht kompliziert und sollte leicht in 10 Minuten auszufüllen sein. Alle Ihre Informationen werden **streng vertraulich** behandelt.

Bitte setzen Sie für jede Frage ein Kreuz „X“ auf die untenstehende Linie, um anzugeben wie schwerwiegend Ihr Schluckproblem ist.
Beispiel:
Sie setzen das „X“ auf der **linken** Seite der Linie, wenn Sie Ihr Problem als **gering** einschätzen, in die **Mitte**, wenn es sich um ein **mittelmässig grosses** Problem handelt und auf die **rechte** Seite der Linie, wenn Ihr Problem sehr **schwerwiegend** ist.
Sie setzen das „X“ **ganz an das linke Ende** der Linie, wenn Sie **kein** Problem haben.

1. Wie gross sind Ihre **Schwierigkeiten in letzter Zeit** beim Schlucken?

überhaupt keine Schwierigkeiten |————————————| absolut unfähig zu schlucken

2. Wie viel Schwierigkeiten haben Sie, **Getränke mit dünnflüssiger Konsistenz** (wie Tee, Softdrinks, Bier oder Kaffee) zu schlucken?

überhaupt keine Schwierigkeiten |————————————| absolut unfähig zu schlucken

3. Wie viel Schwierigkeiten bereitet Ihnen das **Schlucken von <u>dickflüssigen</u> Getränken oder Speisen** (wie Milchshake, Suppe oder Vanillesosse)?

überhaupt keine Schwierigkeiten		absolut unfähig zu schlucken

4. Wie viel Schwierigkeiten bereitet Ihnen das **Schlucken von <u>weichen</u> Nahrungsmitteln** (wie Apfelmus, Rührei oder Kartoffelpüree)?

überhaupt keine Schwierigkeiten		absolut unfähig zu schlucken

5. Wie viel Schwierigkeiten bereitet Ihnen das **Schlucken von <u>festen</u> Nahrungsmitteln** (wie Steak, rohem Apfel oder roher Karotte)?

überhaupt keine Schwierigkeiten		absolut unfähig zu schlucken

6. Wie viel Schwierigkeiten bereitet Ihnen das **Schlucken von <u>trockenen</u> Nahrungsmitteln** (wie Brot, Keksen oder Nüssen)?

überhaupt keine Schwierigkeiten		absolut unfähig zu schlucken

7. Haben Sie Schwierigkeiten Ihren eigenen **Speichel zu schlucken**?

überhaupt keine Schwierigkeiten		absolut unfähig zu schlucken

8. Haben Sie Schwierigkeiten, beim **Hinunterschlucken**?

Niemals		jedes Mal, wenn ich schlucke

9. Haben Sie das Gefühl, dass Essen **in Ihrem Rachen hängen** bleibt?

Niemals		jedes Mal, wenn ich schlucke

10. Müssen Sie jemals **husten oder würgen**, wenn Sie **feste Speisen** (wie Brot, Fleisch oder Äpfel) essen?

Niemals |————————————| jedes Mal, wenn ich esse

11. Müssen Sie **husten oder würgen**, wenn Sie **Flüssigkeiten** (wie Kaffee, Tee, Wasser oder Bier) trinken?

Niemals |————————————| jedes Mal, wenn ich trinke

12. Wie lange benötigen Sie für eine **durchschnittliche Mahlzeit**?
Bitte kreuzen Sie <u>eine</u> Möglichkeit an:
- ☐ Weniger als 15 Minuten
- ☐ Ungefähr 15 - 30 Minuten
- ☐ Ungefähr 30 - 45 Minuten
- ☐ Ungefähr 45 - 60 Minuten
- ☐ Mehr als 60 Minuten
- ☐ Ich kann überhaupt nicht schlucken

13. Gelangen Flüssigkeiten oder Nahrungsmittel beim Schlucken **in die Nase oder kommen aus der Nase heraus?**

Niemals |————————————| jedes Mal, wenn ich schlucke

14. Müssen Sie **mehr als einmal schlucken**, damit das Essen runter geht?

Niemals |————————————| jedes Mal, wenn ich schlucke

15. Müssen Sie **<u>während</u> einer Mahlzeit Nahrungsmittel oder Flüssigkeit hochhusten oder ausspucken**?

niemals |————————————| jedes Mal, wenn ich esse oder trinke

16. Wie hoch schätzen Sie den **Schweregrad Ihres Schluckproblems heute** ein?

kein Problem —————————————————— extrem schwerwiegendes Problem

17. Wie sehr **beeinträchtigt** Ihr Schluckproblem Ihre **Lebensfreude oder Ihre Lebensqualität**?

keine Beeinträchtigung —————————————————— extrem schwere Beeinträchtigung

Besten Dank für Ihre Mitarbeit
